# αB-crystallin and HspB2 deficiency is protective from diet-induced glucose intolerance

**DOI:** 10.1016/j.gdata.2016.03.010

**Published:** 2016-05-13

**Authors:** Daniel J. Toft, Miles Fuller, Matthew Schipma, Feng Chen, Vincent L. Cryns, Brian T. Layden

**Affiliations:** a Division of Endocrinology, Metabolism and Molecular Medicine, Northwestern University Feinberg School of Medicine, Chicago, IL, United States; b Next Generation Sequencing Core, Northwestern University Feinberg School of Medicine, Chicago, IL, United States; c Division of Endocrinology, Diabetes and Metabolism, Department of Medicine, University of Wisconsin School of Medicine and Public Health, Madison, WI, United States; d Jesse Brown Veterans Affairs Medical Center, Chicago, IL, United States

**Keywords:** Glucose tolerance, αB-crystallin, HspB2

## Abstract

Emerging evidence suggests molecular chaperones have a role in the pathogenesis of obesity and diabetes. As αB-crystallin and HspB2 are molecular chaperones and data suggests their expression is elevated in the skeletal muscle of diabetic and obese animals, we sought to determine if αB-crystallin and HspB2 collectively play a functional role in the metabolic phenotype of diet-induced obesity. Using αB-crystallin/HspB2 knockout and littermate wild-type controls, it was observed that mice on the high fat diet gained more weight as compared to the normal chow group and genotype did not impact this weight gain. To test if the genotype and/or diet influenced glucose homeostasis, intraperitoneal glucose challenge was performed. While similar on normal chow diet, wild-type mice on the high fat diet exhibited higher glucose levels during the glucose challenge compared to the αB-crystallin/HspB2 knockout mice. Although wild-type mice had higher glucose levels, insulin levels were similar for both genotypes. Insulin tolerance testing revealed that αB-crystallin/HspB2 knockout mice were more sensitive to insulin, leading to lower glucose levels over time, which is indicative of a difference in insulin sensitivity between the genotypes on a high fat diet. Transcriptome analyses of skeletal muscle in αB-crystallin/HspB2 knockout and wild-type mice on a normal or high fat diet revealed reductions in cytokine pathway genes in αB-crystallin/HspB2 knockout mice, which may contribute to their improved insulin sensitivity. Collectively, these data reveal that αB-crystallin/HspB2 plays a role in development of insulin resistance during a high fat diet challenge.

## 1 Introduction

The αB-crystallin and HspB2 genes reside in a head-to-head orientation encoding small heat shock proteins [1]. An orientation dependent intragenic promoter permits the differential expression of the two genes [1], [2], [3], [4]. αB-crystallin is highly expressed in the crystalline lens and muscle [5]. The αB-crystallin gene (Cryab) encodes a protein that acts as molecular chaperone and cell death antagonist that inhibits caspase-3 activation [6], [7], [8]. In humans, mutations resulting in altered αB-crystallin sequence results in eye diseases including cataracts, as well as in muscle diseases such as myopathies [9], [10], [11]. Not surprisingly, in these diseases, there is evidence of abnormal protein aggregation, likely secondary to diminished chaperone activity of αB-crystallin. HspB2 has been shown to also have chaperone activity [12], [13] and anti-apoptotic activity [14]. p53 has been shown to regulate the expression of both αB-crystallin and HspB2, and silencing expression of αB-crystallin, but not HspB2, in MCF-7 breast cancer cells leads to higher intracellular glucose levels [15]. A genetic knockout (KO) mouse model exists deleting both αB-crystallin and HspB2 genes; these mice develop a progressive myopathy as they age [16].

While its role in cataracts and myopathies is well established, αB-crystallin and HspB2 may also play a pathogenic role in other diseases. Indeed, αB-crystallin transcripts are upregulated in the muscle of diabetic animals [17], [18], [19] and in humans with obesity [18], [20], [21], [22], suggesting that αB-crystallin may contribute to the etiology of these disorders. These observations are particularly interesting because other heat shock proteins have been reported to have a role in obesity and type 2 diabetes, which may reflect their ability to modulate the inflammatory process that occurs with insulin resistance. Hsp72 has been shown by one group to be reduced in skeletal muscle from diabetic subjects with genetic overexpression of Hsp72 improving glucose tolerance in mice [23], [24]. Other studies have found no change in Hsp72 levels in skeletal muscle from diabetic patients but instead found Hsp90 to be overexpressed [25]. Taken together, these data led us to investigate if αB-crystallin/HspB2 may have a role in obesity-related metabolic disorders.

To evaluate the functional role of αB-crystallin/HspB2 in diet-induced obesity and diabetes, we placed αB-crystallin/HspB2-deficient and littermate controls (wild-type, WT) on normal chow or high fat diets and monitored weight and metabolic parameters over time. We rigorously tested their glucose homeostasis *via* glucose tolerance tests and their insulin resistance by insulin tolerance tests throughout these dietary interventions. The outcome revealed that αB-crystallin/HspB2 has a role in the development of glucose intolerance, likely by increasing insulin resistance. Because of this, we profiled the skeletal muscle transcriptome between the genotypes and under each dietary condition. Taken together, these data reveal that αB-crystallin/HspB2 is involved in the genesis of insulin resistance on a high fat diet, and we provide extensive RNA profiling to illuminate potential mechanistic insights into the muscle-specific role of αB-crystallin/HspB2.

## 2 Materials and methods

### 2.1 Animals

αB-crystallin/HspB2 knockout mice [16] in the FVB/N background were kindly provided by Dr. Xuejun Wang (University of South Dakota). By a heterozygous breeding approach, WT and KO male mice were generated. All mice were group housed with a maximum of 5 animals per cage in a temperature controlled facility with 12 hour light-dark cycle and *ad libitum* access to normal chow (LM-485, Harlan Laboratories, Indianapolis, IN) and water. For high fat diet experiments, mice were given *ad libitum* access to high fat chow (TD.06414, Harlan Laboratories, Indianapolis, IN) beginning at 6 weeks of age. Energy density of normal chow diet was 3.1 kcal/g, and 5.1 kcal/g for high fat chow (with approximately 60% of calories from fat). All experiments described herein and this specific study (protocol 2011-2561) were approved by the Institutional Animal Care and Use Committee at Northwestern University. The primary method of euthanasia for the mice was carbon dioxide; once all signs of life were absent cervical dislocation followed as the secondary euthanasia method.

### 2.2 Glucose tolerance test

Blood was obtained from tail veins for glucose determination (measured with a One-Touch Ultra Glucometer) and insulin assays (by ELISA assay, ALPCO). Both oral (OGTT) and intraperitoneal glucose tolerance tests (IPGTT) were done on mice fasted overnight and glucose delivery occurred either by intraperitoneal injection (2.0 g/kg body weight) or oral gavage (2 g/kg body weight). Glucose and/or insulin levels were measured at multiple time points (0–180 min) during the OGTT and IPGTT. For both the IPGTT and OGTT, area under the curve was calculated by standard approaches using the trapezoidal rule.

### 2.3 Insulin tolerance tests

Insulin tolerance tests (ITTs) were conducted on mice that were fasted for 6 h with insulin delivered by intraperitoneal injection at 0.75 U/kg. Glucose was measured at multiple time points from blood obtained over 0–120 min and plotted as percentage of the glucose at 0 min.

### 2.4 Transcriptome analyses

For reasons related to the metabolic phenotype, we explored the transcriptome in skeletal muscle, where αB-crystallin/HspB2 knockout male mice (at 22 weeks of age) were either on normal chow or high fat diet and three independent samples per genotype were used in the analysis. First, the hind leg muscle was isolated at the time the mice were euthanized and immediately frozen in liquid N_2_. Next, the total RNA was extracted with Rneasy Mini Kit (Qiagen) assessed for quality with Agilent 2100 Bioanalyzer. High quality RNA was submitted to Northwestern University Genomic Sequencing Core for sequencing by an Illumina HiSeq2000 using 100-bp paired-end reads. Statistically significant differentially expressed genes in the transcriptome were analyzed using Gene Ontology (GO) analysis and other conventional pathway analyses. The dataset has been deposited at GEO under the accession number GSE66557.

### 2.5 RNA extraction and quantitative real-time PCR

Total cellular RNA was extracted from skeletal muscle tissue from αB-crystallin/HspB2 KO or WT mice (*n* = 3, male mice at 22 weeks of age) and homogenized in Trizol (Invitrogen) using an RNeasy Mini kit (QIAGEN). Quantitative real-time PCR was performed using a 1-Step SYBR Green qRT-PCR Kit. The relative expression of the target genes was determined by the comparative ΔCt method after normalization to β-actin. Primer sequences are available on request.

### 2.6 Protein extraction and western blotting

Skeletal muscle tissue from the mouse models (*n* = 3) was lysed with 250 μl of RIPA lysis buffer including protease (Thermo Scientific #87786) and phosphatase inhibitor cocktails (Thermo Scientific #78420). Samples were homogenized using a handled polytron for 30 s, incubated on ice for 30 min, and then centrifuged at 15,000 ×* g* for 15 min at 4 °C to remove cell debris. Total protein was measured using a Micro BCA protein Assay (Thermo Scientific #23235). 20 μg of protein was loaded into an 8% SDS PAGE gel and transferred onto a nitrocellulose membrane. The following antibodies were used: αB-crystallin (Enzo Life Technologies; ADI-SPA-222-D, 1:1000), HSP B2 (Santa Cruz; sc-292205, 1:200), α-Tubulin (Sigma; T5168, 1:4000), anti-Mouse IgG HRP conjugate (Promega; W402B, 1:7000) and anti-Rabbit IgG HRP conjugate HRP conjugate (Promega; W401B, 1:7000). Amersham ECL Western Blotting Detection Reagents (GE Healthcare; RPN2106) was used to detect chemiluminescence with HyBlot CL autoradiography film (Denville Scientific Inc.).

### 2.7 Statistical analyses

Values are reported as the mean ± SEM unless otherwise indicated. *p* values were calculated by Student's *t*-test (two-tailed, unpaired) with a significance level at *p* < 0.05 unless otherwise indicated.

## 3 Results

### 3.1 Metabolic phenotyping

**Fig. 1:**
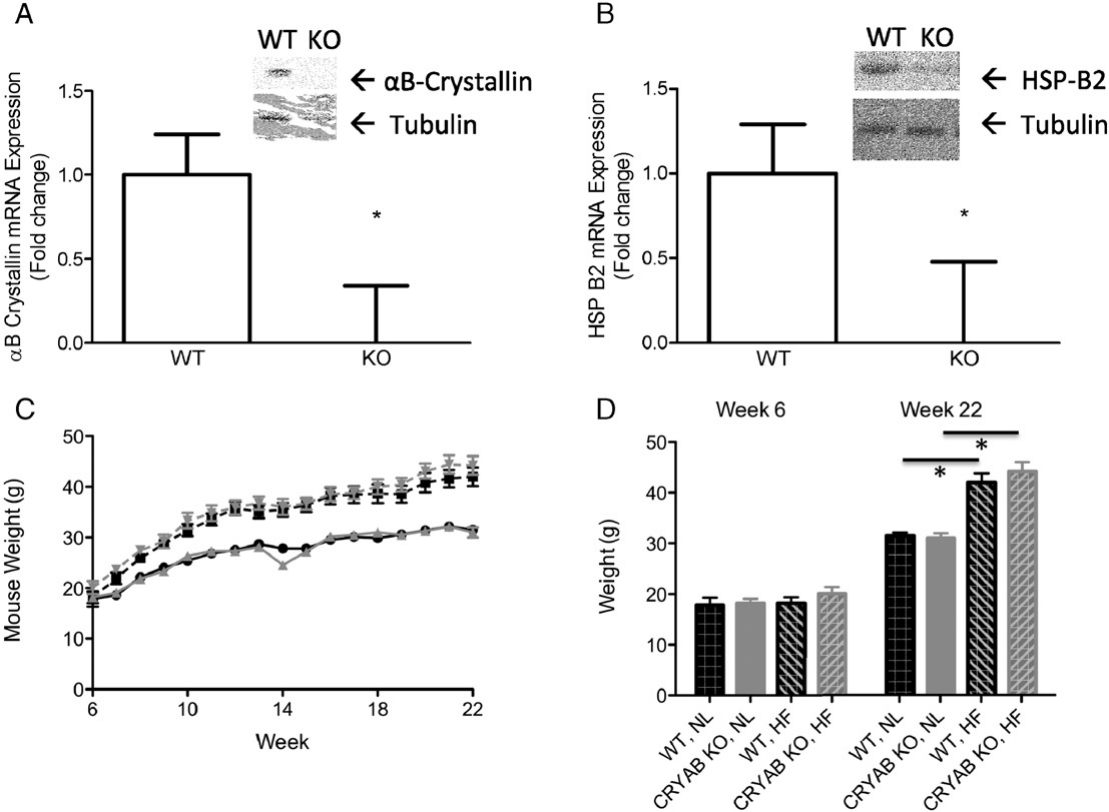
Weight changes to αB-crystallin/HspB2 KO and WT mice. A–B) Real-time PCR (*n* = 3 from independent replicates, mean ± SEM) and Western blot (similar results for 3 independent replicates) evidence of genetic deletion of αB-crystallin and HspB2 is shown, respectively. C) Weekly weights are shown for mice, starting at 6 weeks of age, were followed to week 22, while on normal chow or high fat diet. Shown is mean ± SEM, *n* = 9–11. Black circle-WT mice on normal chow, black square-WT mice on high fat, grey triangle-KO mice on normal chow, and grey-upside triangle on high fat. D) Weights at the start (week 6) and end (week 22) of the experimental study between the diet groups (normal chow *vs.* high fat) and genotypes (αB-crystallin/HspB2 KO *vs.* WT mice). Shown is mean ± SEM, *n* = 9–11. Black bar - WT mice on normal chow, black bar with lines - WT mice on high fat, grey bar - KO mice on normal chow, and grey bar with lines upside triangle on high fat. *, *p* < 0.05 determined by Student's *t*-test.

Using αB-crystallin/HspB2 knockout (KO) and matched littermate wild-type (WT) male mice (evidence of genetic deletion of each gene is shown in Fig. 1A–B), we first assessed their weight gain over time on a normal chow diet and also on a diet that induced weight gain and impaired glucose tolerance (high fat diet, 60% kcals of fat). Beginning at week 6 of age, we followed their weights over time. Mice on a high fat diet rapidly gained more weight independent of genotype as compared to the normal chow group (Fig. 1C). Importantly, the genotype did not influence weight gain over time between WT and KO mice on normal chow or high fat diets during the period investigated (Fig. 1D). These data indicate that global ablation of αB-crystallin/HspB2 is not a factor in weight gain on either diet.

**Fig. 2:**
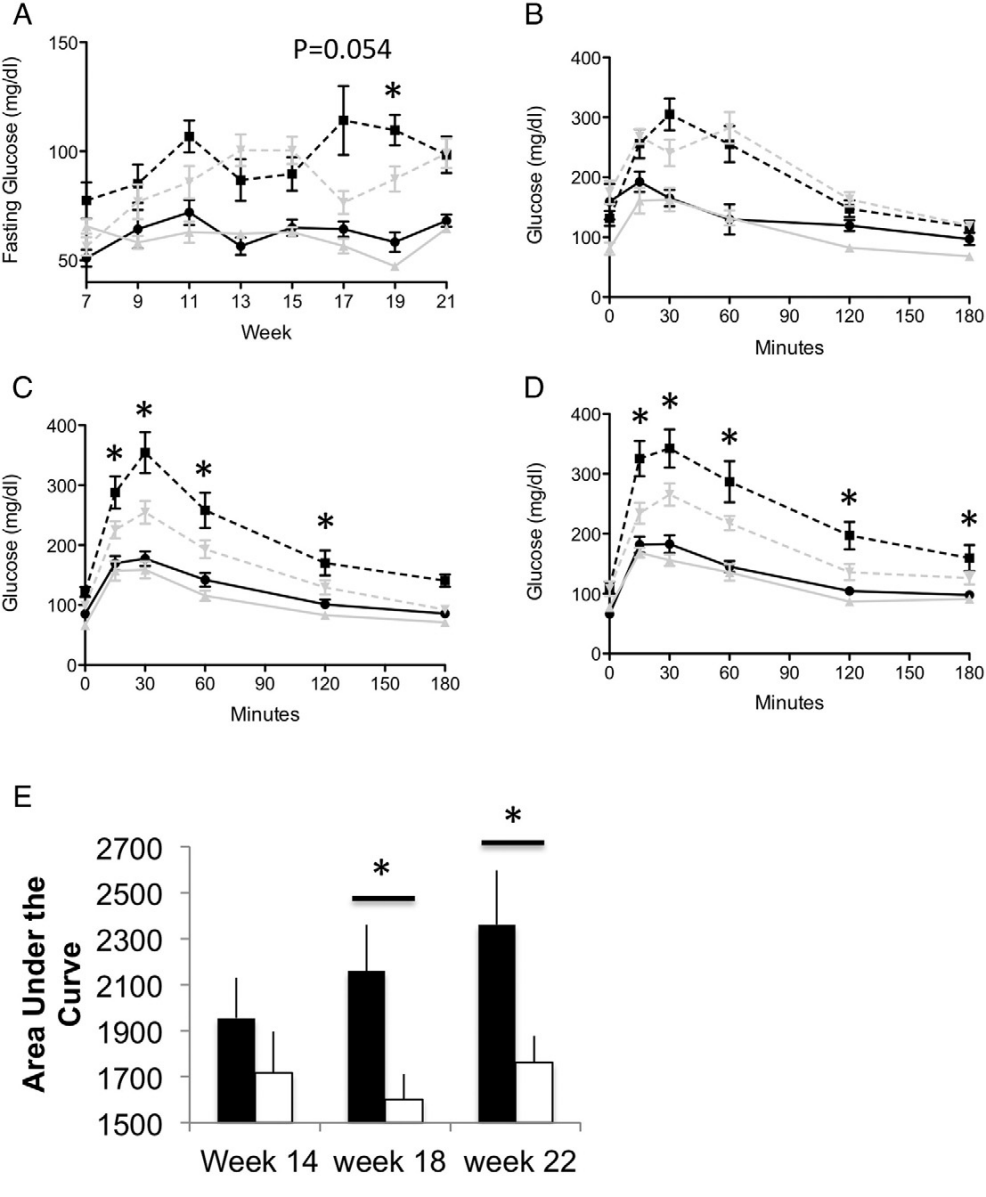
Glucose levels and glucose tolerance for αB-crystallin/HspB2 KO and WT mice. A) Fasting glucose levels shown for mice, starting at 6 weeks of age, followed to week 22, while on normal chow or high fat diet. Shown is mean ± SEM, *n* = 9–11. B–D) Glucose levels during a glucose tolerance challenge delivery by intraperitoneal injection at week 14 (B), 18 (C), and 22(D). Black circle-WT mice on normal chow, black square-WT mice on high fat, grey triangle-KO mice on normal chow, and grey-upside triangle on high fat. E) Area under the curve calculated at 14, 18, and 22 weeks of age between WT (black bar) and KO (clear bar) mice on high fat diet. *, *p* < 0.05.

To begin to assess glucose homeostasis over time during the dietary intervention, we measured fasting glucose at multiple points during this period. Between the two dietary groups, the group of mice on high fat diet, regardless of genotype had higher fasting glucose levels. However, no major difference was apparent between the groups except at week 19 in the high fat group, where the KO mice had lower fasting glucose compared to the WT mice (Fig. 2A). To more rigorously explore glucose homeostasis between the genotypes, glucose tolerance throughout this period was assessed by intraperitoneal glucose challenge. Notably, no difference in glucose tolerance throughout this time period occurred between WT and KO mice on normal chow diets (data not shown). On the high fat diet, a progressive phenotype emerged between the genotypes in the high fat group (Fig. 2B–E). Specifically, at 14 weeks of age (8 weeks of high fat diet), no difference was apparent between WT and KO mice on high fat diet during the IPGTT challenge (Fig. 2B). At week 18 and 22 (12 and 16 weeks of high fat diet), the WT mice had higher glucose levels during the glucose challenge compared to the KO mice starting at time 15 min (except for one time point at 180 min at week 18, Fig. 2C) (Fig. 2C–D). Furthermore, the area under the curve was progressively less in the KO as compared to the WT mice (Fig. 2E) at 18 and 22 weeks. Taken together, these data indicate the WT mice develop impaired glucose tolerance, which is the expected response on a high fat diet; however, the KO mice do not develop this glucose impairment to the same degree.

**Fig. 3:**
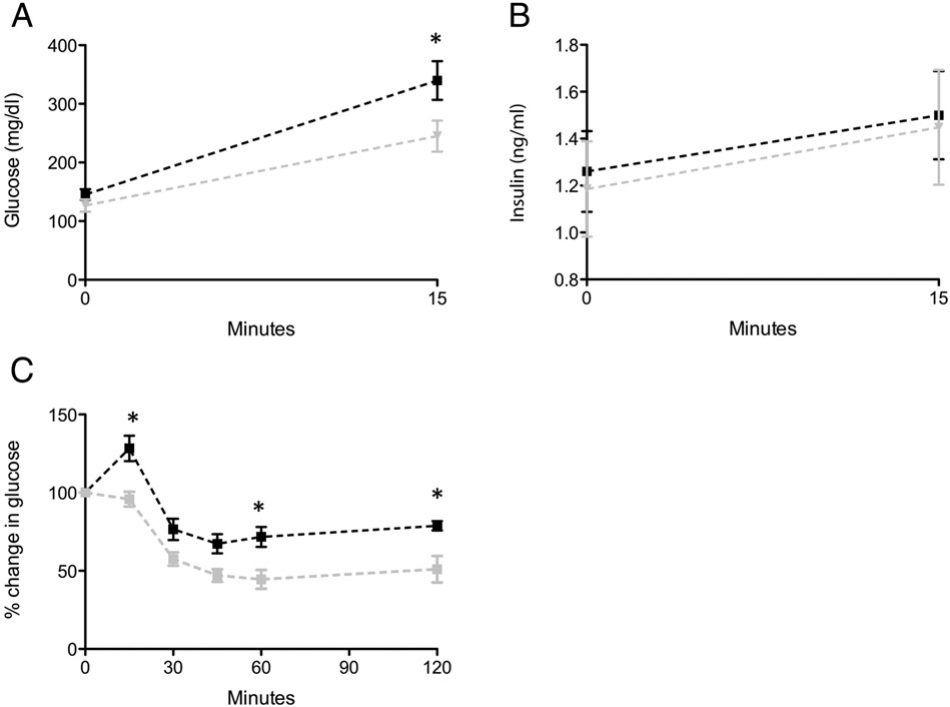
*In vivo* insulin secretion and insulin tolerance for αB-crystallin/HspB2 KO and WT mice at 22–24 weeks of age. A–B) Glucose and insulin levels shown for mice on a high fat diet during an intraperitoneal glucose challenge at 0 and 15 min. Shown is mean ± SEM, *n* = 6–8. Black line-WT, grey line-KO. C) Insulin tolerance test for mice on a high fat diet, where percentage change in glucose from time 0 is shown, mean ± SEM, *n* = 6–9. Black line-WT, Grey line-KO. *, *p* < 0.05.

Next, we investigated if insulin secretion and/or insulin sensitivity contributed to the observed differences in glucose tolerance in these studies. First, to assess insulin secretion, the mice were again challenged with intraperitoneal glucose, and glucose and insulin levels were assessed at time 0 and 15 min. As seen in Fig. 3A–B, the WT mice had higher glucose levels than the KO mice at time 15 min, consistent with the glucose levels seen in Fig. 2, but the insulin levels, at time 0 and 15 min, were similar, suggesting no major difference in insulin secretion. To assess whether alterations in insulin sensitivity were accounting for the impaired glucose tolerance, an insulin tolerance test was also performed. The KO mice were more sensitive to insulin, leading to lower glucose levels over time (Fig. 3C). Cumulatively, these data suggest that αB-crystallin/HspB2 KO mice on a high fat diet have improved glucose tolerance, which may be a result of diminished insulin resistance.

### 3.2 Transcriptome analyses

αB-crystallin and HspB2 are highly expressed in skeletal muscle [5], a likely tissue where αB-crystallin/HspB2 activity may affect insulin resistance, especially considering αB-crystallin is upregulated in muscle in rodents and humans in the setting of insulin resistance [18], [19], [20]. Therefore, we performed transcriptome analyses of the proximal hind leg muscle of KO and WT mice on normal and high fat diets.

**Fig. 4:**
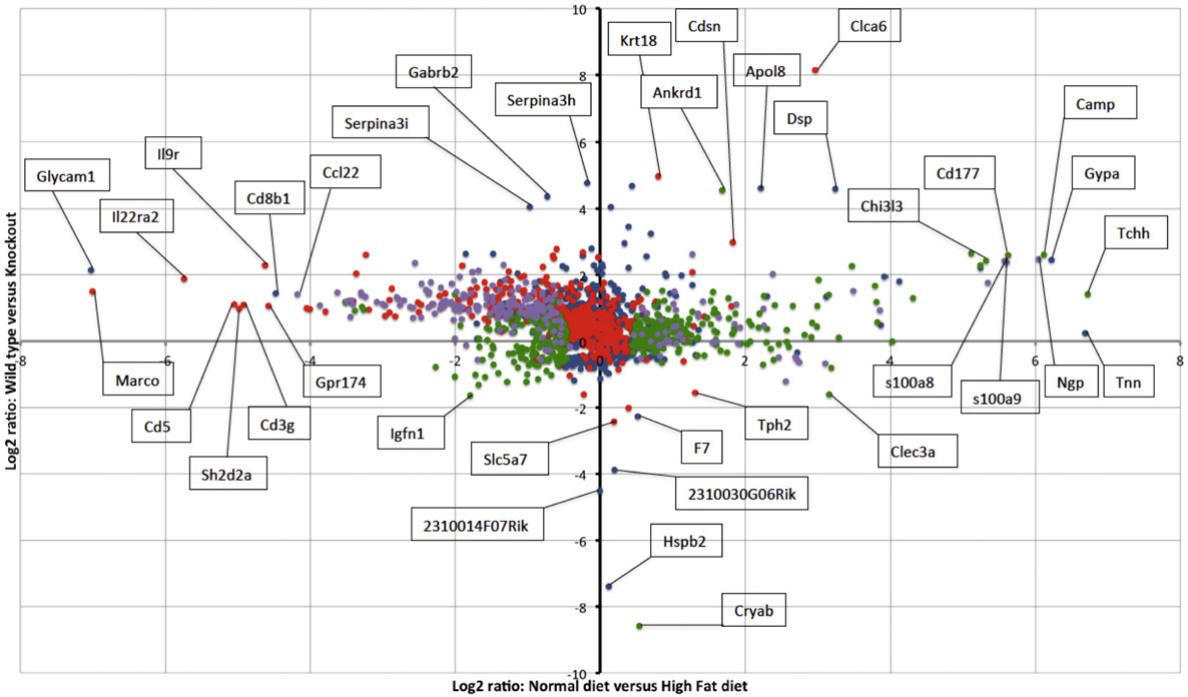
Log (base 2) ratios of gene expression intensities in αB-crystallin/HspB2 KO and WT mice on the high fat diets and control diets. The y-axis shows the comparison based on genotype. The x-axis compares high fat and control diets regardless of strain. Red dots represent genes that are significantly differentially expressed in αB-crystallin/HspB2 KO on high fat diet compared to control diet. Green dots represent genes that are significantly differentially expressed in αB-crystallin/HspB2 WT mice on high fat diet compared to control diet. Purple dots represent genes that are significantly differentially expressed in αB-crystallin/HspB2 KO and WT mice on high fat diet compared to control diet. Several genes of interest are marked and labeled.

In supplemental file 1 and Table 1, Table 2, Table 3, Table 4, the differentially regulated genes are presented, based on thresholds described in the Methods. There were 645 differentially regulated genes in WT mice between the dietary conditions (Table 1, top genes up and down regulated shown). Many keratin and keratin related proteins were strongly upregulated by high fat diet; interestingly αB-crystallin was significantly upregulated in high fat animals, while HspB2 expression was not significantly different. Moreover, there were 737 differentially regulated genes in KO mice between the dietary conditions (Table 2, top genes up and down regulated shown). Interestingly, genetic KO of αB-crystallin/HspB2 led to no alterations in keratin and keratin-related proteins. In addition, there were 938 differentially regulated genes between KO and WT mice maintained on normal chow (Table 3, top genes up and down regulated shown). Of interest, a few keratin genes were altered compared to KO mice. Finally, there were 1055 differentially regulated genes between KO and WT mice maintained on a high fat diet (Table 4, top genes up and down regulated shown). To examine this data in another manner (Fig. 4), we plotted the summary of these data, through log (base 2) ratios of gene expression intensities in αB-crystallin/HspB2 KO and WT mice on high fat and control diets.

Additionally, we evaluated the collective transcript changes based on pathway analysis through KEGG pathway database (Tables S1–S4). Of interest, the effect of high fat diet induced higher expression of genes within the following classes (primary immunodeficiency, cell adhesion molecules, T cell receptor signaling, cytokine-cytokine receptor interaction, and insulin signaling), which was independent of genotype (Table S1 and S2). The effect of genotype for mice on a normal diet (Table S3) only resulted in downregulated class of genes in KO mice, where one of these classes (cytokine-cytokine receptor interaction) was also observed to be induced by high fat diet in KO mice. And finally, the high fat diet induced a greater response of genes involved in fatty acid metabolism in the KO mice, as compared to WT mice, on high fat diet (Table S4).

## 4 Discussion

We have demonstrated that genetic ablation of αB-crystallin/HspB2 results in improved glucose tolerance and diminished insulin resistance in mice maintained on a high fat diet. These data implicate αB-crystallin/HspB2 in the pathogenesis of diet-induced diabetes and are particularly intriguing in light of multiple studies suggesting that insulin resistance is associated with higher levels of αB-crystallin expression in skeletal muscle [17], [18], [19], [20], [21], [22], [26]. Our data suggest that αB-crystallin/HspB2 may contribute to insulin resistance through its role in skeletal muscle. However, because these are global KO mice, other tissues such as islets and adipose tissue may be contributing to this phenotype. Moreover, we cannot determine if αB-crystallin, HspB2 or both are the relevant gene(s) involved in the observed effects given the limitation of our combined αB-crystallin/HspB2 KO model. To begin to identify the underlying molecular mechanisms, we extensively profiled the transcript changes based on genotype (αB-crystallin/HspB2 KO or WT) and diet (normal or high fat diet).

Insulin resistance is an inflammatory state, which is in part mediated by increased cytokine signaling [27]. KEGG pathway analysis revealed that cytokine-cytokine receptor pathways and insulin signaling were upregulated by the high fat diet in the WT mice (Table S1) and KO mice (Table S2). Surprisingly, only a few studies have been performed examining the effects of high fat diet on the transcriptome of skeletal muscle in mice, and the ones that have been published have examined mice with different genetic backgrounds using different high fat diets than the one used in this study, and the duration of the high fat diet was overall much shorter than used by us [28], [29], [30]. Thus, it is not feasible to perform an extensive comparison to these studies. However, on normal chow and high fat diet, KO mice had fewer cytokine-cytokine receptor pathway genes altered (Tables S3–S4). As cytokine signaling induces cellular stress and αB-crystallin/HspB2 both contribute to this response [31], [32], [33], αB-crystallin/HspB2 may directly link inflammation and insulin resistance. This observation is notable as αB-crystallin and HspB2 are generally thought to be protective against some forms of cellular inflammation such as redox stress [34]. However, deficiency of αB-crystallin and HspB2 has been observed to result in protection from myocardial ischemia in some studies [35], which was also unexpected per these authors, and was suggested to be from altered mitochondrial metabolism. In a subsequent study the authors found that animals harboring a cardiac-specific HspB2 genetic deletion had an equal propensity for ischemia induced cardiac hypertrophy suggesting that αB-crystallin and HspB2 have at least partly redundant activities in the heart [36]. Moving forward, the next steps will be to determine the mechanisms by which αB-crystallin or HspB2 individually and collectively regulate insulin resistance in muscle.

Interestingly, heat shock proteins have been shown to be mediators of insulin sensitivity in skeletal muscle [37]. Specifically, the loss of heat shock proteins is associated with aging and insulin resistance [37]. In contrast, we observed that deletion of αB-crystallin/HspB2 in mice led to improved insulin sensitivity. Considering the alteration of cytokine-pathways we observed in the transcriptome analyses, these data, nonetheless, suggest a potential role of αB-crystallin/HspB2 in cytokine induced oxidative stress. Taken together, we provide evidence that genetic KO of αB-crystallin/HspB2 leads to less insulin resistance while mice are on a high fat diet. Our transcript profiling provides a foundation to begin to decipher the molecular mechanisms by which αB-crystallin and/or HspB2 mediates insulin resistance.

## Conflict of interest

The authors have nothing to disclose.

## Figures and Tables

**Table 1:** Top ten genes up and down regulated comparing for WT mice on high fat diet *vs.* normal chow diet.^a^

Gene		Fold change	*p* value
Apoa2	NM_013474	Inf	0.00148154
Bglap2	NM_001032298	Inf	0.00148154
Crabp1	NM_013496	Inf	0.00148154
Ear6,Ear7	NM_017385	Inf	0.00148154
Gm10229	NM_001199334	Inf	0.00148154
Gm5483	NM_001082547	Inf	0.00148154
Krt25	NM_133730	Inf	0.00148154
Krt27	NM_010666	Inf	0.00148154
Krt28	NM_027574	Inf	0.00148154
Krt31	NM_010659	Inf	0.00148154
Krt33a	NM_027983	Inf	0.00148154
Krt34	NM_027563	Inf	0.00148154
Krt71	NM_019956	Inf	0.00148154
Krt72-ps	NM_213728	Inf	0.00148154
Krt73	NM_212485	Inf	0.00148154
Krt77	NM_001003667	Inf	0.00148154
Krtap14	NM_013707	Inf	0.00148154
Krtap16-10b	NM_133359	Inf	0.00148154
Krtap16-8	NM_130856	Inf	0.00148154
Krtap22-2	NM_001191018	Inf	0.00869015
Krtap3-1	NM_023511	Inf	0.00148154
Krtap3-3	NM_025524	Inf	0.00148154
Krtap7-1	NM_027771	Inf	0.00148154
Krtap8-1	NM_010675	Inf	0.00148154
Lgals7	NM_008496	Inf	0.00148154
Mcpt8	NM_008572	Inf	0.00148154
Prss34	NM_178372	Inf	0.00148154
Stfa2l1	NM_173869	Inf	0.00148154
Trem3	NM_021407	Inf	0.00148154
Tnfrsf13c	NM_028075	− 3.38526	0.0151089
Itk	NM_010583	− 3.41527	0.00387886
Il21r	NM_021887	− 3.43414	0.00148154
Icos	NM_017480	− 3.44552	0.0151089
Gimap3	NM_031247	− 3.49913	0.00148154
Faim3	NM_026976	− 3.51499	0.00275481
Ccr7	NM_007719	− 3.62644	0.00491158
Cd3e	NM_007648	− 3.86742	0.00491158
Ccl22	NM_009137	− 4.17435	0.00956225
H2-M2	NM_008204	-Inf	0.00148154

a More than 10 genes are provided if over 10 genes had infinite changes.

**Table 2:** Top ten genes up and down regulated comparing for KO mice on high fat diet *vs.* normal chow diet.

Gene		Fold change	*p* value
Snora31	NR_028481	Inf	0.0458367
Mmp12	NM_008605	5.76031	0.00148154
Slc5a7	NM_022025	4.92492	0.00148154
Ubd	NM_023137	4.78419	0.00148154
Tph2	NM_173391	4.63977	0.00148154
1700047G03Rik	NR_040447	4.35806	0.00590586
Cdkl4	NM_001033443	4.14468	0.0229947
Oxtr	NM_001081147	3.92615	0.00148154
Atp6v0d2	NM_175406	3.65238	0.00148154
Ncan	NM_007789	3.50117	0.00148154
Ikzf3	NM_011771	− 5.59252	0.00275481
Ltb	NM_008518	− 5.6414	0.00148154
Pou2af1	NM_011136	− 5.77367	0.00148154
Cd19	NM_009844	− 5.85446	0.00148154
Ms4a1	NM_007641	− 6.05748	0.00387886
Marco	NM_010766	− 6.08431	0.00148154
Slpi	NM_011414	− 6.71785	0.00275481
Cd40lg	NM_011616	-Inf	0.00148154
Dapl1	NM_029723	-Inf	0.00148154
Tspan10	NM_145363	-Inf	0.00148154

**Table 3:** Top ten genes up and down regulated comparing WT *vs.* KO mice on normal chow.

Gene		Fold change	*p* value
Ripply1	NM_001037915	Inf	0.00148154
Krt18	NM_010664	4.9788	0.00148154
Serpina3h	NR_033450	4.77386	0.00148154
Krt8	NM_031170	4.68042	0.0364368
Dsp	NM_023842	4.59612	0.00148154
Ankrd1	NM_013468	4.54928	0.00148154
Gabrb2	NM_008070	4.36359	0.00148154
Serpina3i	NM_001199940	4.05357	0.00869015
Mmel1	NM_013783	4.05285	0.0375712
Hsf2bp	NM_028902	3.45121	0.00148154
Dixdc1	NM_178118	− 1.12517	0.0195563
Vipr2	NM_009511	− 1.14549	0.00148154
Itgb1bp3	NM_027120	− 1.17642	0.00148154
Has3	NM_008217	− 1.22099	0.00781388
Prnd	NM_001126338	− 1.22638	0.00956225
Cacna2d4	NM_001033382	− 1.31364	0.00148154
Igfn1	NM_177642	− 1.63024	0.00148154
2310030G06Rik	NM_025865	− 3.88292	0.00148154
2310014F07Rik	NR_040714	− 4.51427	0.0305894
Hspb2	NM_001164708	− 7.38569	0.00148154

**Table 4:** Top ten genes up and down regulated comparing WT *vs.* KO mice on high fat diet.^a^

Gene		Fold change	*p* value
Ripply1	NM_001037915	Inf	0.00148154
Snora31	NR_028481	Inf	0.0458367
Serpina3i	NM_001199940	5.071	0.0398075
Serpina3h	NR_033450	4.97688	0.00148154
Clca6	NM_207208	4.29562	0.00148154
Gabrb2	NM_008070	4.23027	0.00148154
Ear11	NM_053113	4.05628	0.0381342
Ankrd1	NM_013468	3.30478	0.00148154
Mrgprg	NM_203492	3.25541	0.0128482
A930003A15Rik	NR_015488	3.20431	0.00148154
BC100530	NM_001082546	-Inf	0.00148154
Gm10229	NM_001199334	-Inf	0.00148154
Gm5483	NM_001082547	-Inf	0.00275481
Krt15	NM_008469	-Inf	0.00148154
Krt27	NM_010666	-Inf	0.00148154
Krt33a	NM_027983	-Inf	0.00148154
Krt34	NM_027563	-Inf	0.00148154
Krt5	NM_027011	-Inf	0.00148154
Krt72-ps	NM_213728	-Inf	0.00148154
Krt73	NM_212485	-Inf	0.00148154
**Krt77**	NM_001003667	-Inf	0.00148154
Krtap14	NM_013707	-Inf	0.00148154
Krtap15	NM_013713	-Inf	0.00148154
Krtap16-10b	NM_133359	-Inf	0.00148154
Krtap16-3	NM_183296	-Inf	0.00148154
Krtap22-2	NM_001191018	-Inf	0.0136488
Krtap3-1	NM_023511	-Inf	0.00148154
Krtap3-2	NM_025720	-Inf	0.00148154
Krtap3-3	NM_025524	-Inf	0.00148154
Krtap7-1	NM_027771	-Inf	0.00148154
Krtap8-1	NM_010675	-Inf	0.00148154
Lgals7	NM_008496	-Inf	0.00148154
Mcpt8	NM_008572	-Inf	0.00148154
Prss34	NM_178372	-Inf	0.00148154
Stfa2l1	NM_173869	-Inf	0.00148154

a More than 10 genes are provided if over 10 genes had infinite changes.

## Appendix A Supplementary data

Supplementary tables Image 1
