# Supplementary material for: αB-crystallin and HspB2 deficiency is protective from diet-induced glucose intolerance
Source: Genom Data. 2016 May 13;9:10–7. doi: 10.1016/j.gdata.2016.03.010 (PMC4909821; doi:10.1016/j.gdata.2016.03.010)
Supplement: Supplementary file 1 — Supplementary tables [file mmc1.pdf]

Supplemental Data

Table S1. Pathway analysis of αB-crystallin/Hsp2 WT normal *vs.* high fat diet

**Higher expression in obese animals**	**# genes**	**Hyp_c**
Primary immunodeficiency	12	1.22E-14
Cell adhesion molecules (CAMs)	17	9.51E-13
T cell receptor signaling annotation	15	2.24E-12
Tuberculosis	16	1.46E-10
Cytokine-cytokine receptor interaction	17	2.29E-09
Intestinal immune network for IgA production	9	3.05E-09
Staphylococcus aureus infection	9	1.10E-08
B cell receptor signaling annotation	10	2.26E-08
Insulin signaling annotation	12	4.21E-08
Phagosome	13	4.30E-08

**Lower expression in obese animals**		
Fatty acid metabolism	10	1.89E-10
ECM-receptor interaction	12	1.95E-10
Focal adhesion	14	2.40E-08
Valine, leucine and isoleucine degradation	7	2.79E-06
Fatty acid elongation in mitochondria	4	5.21E-06
Protein digestion and absorption	7	4.72E-05
Propanoate metabolism	5	6.03E-05
PPAR signaling annotation	6	3.88E-04
Cardiac muscle contraction	6	3.89E-04
Peroxisome	6	4.01E-04

Table S2. Pathway analysis of αB-crystallin/HspB2 KO normal *vs.* high fat diet

**Higher expression in obese animals**	**# genes**	**Hyp_c**
Cell adhesion molecules (CAMs)	30	8.20E-24
Hematopoietic cell lineage	18	9.09E-15
T cell receptor signaling annotation	20	9.75E-15
Primary immunodeficiency	13	3.43E-14
Cytokine-cytokine receptor interaction	26	1.25E-13
Chemokine signaling annotation	21	5.70E-12
Leukocyte transendothelial migration	17	3.06E-11
Insulin signaling annotation	18	3.47E-11
Jak-STAT signaling annotation	18	2.33E-10
Viral myocarditis	14	2.93E-10
**Lower expression in obese animals**		
Lysosome	8	4.52E-06
Rheumatoid arthritis	7	8.60E-06
Phagosome	7	3.19E-04
Hypertrophic cardiomyopathy (HCM)	5	8.34E-04
Osteoclast differentiation	5	3.45E-03
Complement and coagulation cascades	4	5.93E-03
Dilated cardiomyopathy	4	8.84E-03
Malaria	3	1.13E-02
Tuberculosis	5	1.18E-02
Arginine and proline metabolism	3	1.49E-02

Table S3. Pathway analysis of αB-crystallin/HspB2 WT *vs.* KO normal diet

Higher expression in KO animals	# genes	Hyp_c
No significant enrichment		
**Lower expression in KO animals**		
Cytokine-cytokine receptor interaction	33	3.30E-17
Staphylococcus aureus infection	18	4.76E-17
Phagosome	28	7.16E-17
Cell adhesion molecules (CAMs)	25	9.29E-16
PPAR signaling pathway	19	1.03E-14
Chemokine signaling pathway	24	1.01E-12
Antigen processing and presentation	16	1.00E-11
B cell receptor signaling pathway	16	1.10E-11
Viral myocarditis	16	5.14E-11
Leukocyte transendothelial migration	18	1.11E-10

Table S4 Pathway analysis of αB-crystallin/HspB2 WT *vs.* KO high fat diet

**Higher expression in KO animals**	**# genes**	**Hyp_c**
Fatty acid metabolism	9	3.52E-09
Focal adhesion	13	1.39E-07
ECM-receptor interaction	9	4.72E-07
Valine, leucine and isoleucine degradation	7	1.73E-06
Cardiac muscle contraction	7	2.99E-05
Propanoate metabolism	5	5.21E-05
Oxidative phosphorylation	8	7.32E-05
Parkinson's disease	8	7.90E-05
Bladder cancer	5	1.47E-04
Bile secretion	6	1.61E-04
**Lower expression in KO animals**		
Phagosome	30	5.44E-18
Staphylococcus aureus infection	18	2.16E-16
Complement and coagulation cascades	20	2.13E-15
Tuberculosis	26	7.69E-14
Cytokine-cytokine receptor interaction	30	1.14E-13
Protein digestion and absorption	18	5.01E-13
Osteoclast differentiation	21	5.05E-13
Focal adhesion	26	1.18E-12
ECM-receptor interaction	18	2.06E-12
Amoebiasis	20	2.76E-12
